# Impact of COVID-19 pandemic responses on tuberculosis incidence: insights from Shantou, China

**DOI:** 10.1186/s12889-024-18956-2

**Published:** 2024-05-30

**Authors:** Yaqian Su, Qiaocheng Chang, Ruiming Chen, Zhuanghao Chen, Jianxiong Lin, Hui Fu, Zicheng Cao, Liping Li, Suyang Liu

**Affiliations:** 1 School of Public Health, Shantou University, 234 Daxue Road, Shantou, 515063 China; 2 Shantou Tuberculosis Prevention and Control Institute, Shantou, China

**Keywords:** Tuberculosis, Interrupted time series, COVID-19, Intervention, Pandemic, Quarantine, Lockdown

## Abstract

**Background:**

Various measures taken against the COVID-19 pandemic are not only effective in reducing the spread of the disease, but also lead to some unexpected results. This article regarded these measures as an intervention and explored their impact on the incidence of tuberculosis in Shantou, China.

**Methods:**

The incidence rate and the surveillance data of tuberculosis from January 1st, 2018 to December 31st, 2021 were provided by the Shantou Tuberculosis Prevention and Control Institute. Data were divided into pre-pandemic period (January 1st, 2018 - December 31st, 2019) and pandemic periods (January 1st, 2020 - December 31st, 2021). The Interrupted Time Series (ITS) was used to analyze the trend of tuberculosis incidence prior to and during the COVID-19 epidemic.

**Results:**

The results showed that the incidence of tuberculosis cases in Shantou decreased significantly (*p* < 0.05) during the pandemic as compared to that prior to the pandemic. Among them, the 45–64 age group and the 65 + age group have statistically significant declines. When patients were stratified by occupation, the unemployed and those working in agriculture reduced the most.

**Conclusions:**

In response to the pandemic, measures like lockdowns and quarantines seem to have reduced tuberculosis incidence. However, this does not imply a true decrease. Underlying causes for the reduced true incidence need further scrutiny. Findings offer a preliminary exploration of interventions designed for one disease but functioning as unexpected results for another.

**Supplementary Information:**

The online version contains supplementary material available at 10.1186/s12889-024-18956-2.

## Introduction

The long-standing tuberculosis epidemic and the recent coronavirus disease 2019 (COVID-19) pandemic are important global public health challenges. Tuberculosis is a chronic infectious disease caused by Mycobacterium tuberculosis (MTB) [1]. Tuberculosis can be asymptomatic in its early stages, but as the disease progresses, patients may experience symptoms such as productive cough, hemoptysis, and low-grade fever [2]. The incubation period of chronic pulmonary tuberculosis in some patients can extend to 2–3 months. MTB is mainly transmitted by droplet spread [3]. Individuals with compromised immune systems, diabetes, and those aged 65 years and older are at a higher risk of contracting tuberculosis [4]. Although 90% of tuberculosis patients can be cured, the disease can still seriously endanger one’s health by invading the patient’s organs through blood, lymph or other channels, if not treated or treated improperly. China is one of the countries with the highest burden of tuberculosis. According to a report from the World Health Organization (WHO), there are about 9.9 million new tuberculosis patients by 2020 [5]. Despite a downward trend since 2000, the mortality rate of tuberculosis has been on the rise.

The COVID-19 pandemic has attracted global attention and significantly impacted national healthcare systems in various countries [6–8]. The American Hospital Association estimated that low and middle-income countries may need approximately $52 billion per month (equivalent to $8.60 per person) to provide an effective health care response to the pandemic measures [9]. Studies have shown that the COVID-19 has posed significant challenges to the control of various infectious diseases, including tuberculosis [10, 11]. Measures applied to cope with the pandemic can be viewed as a population-level intervention that may have an important impact on reducing or increasing tuberculosis incidence. Current research in COVID-19 and tuberculosis is centered on examining the interplay between the two diseases, exploring innovative approaches to diagnosis and treatment, assessing susceptibility factors, identifying potential targets for intervention, and so on. Few studies have regarded the response to COVID-19 as an intervention, and assessed its impact on tuberculosis.

Population intervention, or commonly referred to as the group intervention, may accidentally produce an intervention effect on a certain disease, even if it is not designed in the context of the health system. Some well-known examples of population intervention studies include the evaluation of “Olympic Blue” and “APEC Blue”. The term “APEC blue” describes the phenomenon that the sky appears blue following the implementation of a comprehensive air pollution control initiative during the Asia-Pacific Economic Cooperation (APEC) summit held in Beijing in 2014. By comparing environmental data during the APEC summit and non-summit periods, it was found that the measures implemented were successful in improving air quality [12]. Several studies, both in China and other countries, have examined how the preventive measures implemented during the COVID-19 pandemic have affected the incidence of certain infectious diseases, which is similar to our study. For example, a Chinese study found that the incidence of some common airborne diseases, such as seasonal influenza, tuberculosis, and hand-foot-and-mouth disease has dropped significantly after the outbreak of the pandemic [13]. A study in Singapore compared indicators of the influenza activity in 2020 following public health measures in response to COVID-19 with corresponding indicators in the previous three years and found a significant drop in influenza activity [14]. Meanwhile, a Japanese study reported low incidences of some common infectious diseases during the pandemic, perhaps due to containment measures against COVID-19 [15]. The main conclusion of the above three studies is to attribute influenza to the prevention and control measures implemented during the pandemic. By the same token, we also speculate that policies implemented during the pandemic also led to unintended consequences for other diseases, tuberculosis in our case.

Guangdong Province is a high-incidence area for tuberculosis in China, with Shantou city exhibiting particularly elevated rates. Taking the 2022 tuberculosis report for Guangdong as an example, the incidence rate reached 42.56 cases per 100,000 people [16]. Notably, in the past two years, Shantou city had an incidence rate of approximately 59 cases per 100,000 people, surpassing the provincial average. Therefore, conducting an investigation into the incidence and contributing factors of tuberculosis in Shantou city holds significant public health implications for Guangdong Province and the entire country of China.

In our study, we investigated the impact of the COVID-19 pandemic on tuberculosis prevention and control in Shantou, China, by analyzing the changes in tuberculosis incidence rates prior to and during the pandemic. Our research has yielded robust epidemiological evidence, which can be utilized for devising comparable intervention strategies in the future, aiming to prevent any counterfeit or adverse effects that may arise during future pandemics similar to the outbreak of COVID-19.

## Methods

### Study area

Shantou (116°14′-117°19′ North, 23°02′-23°38′ East) is a city situated in the southeastern region of mainland China. As an important transportation hub, it plays a significant role in connecting Guangdong, Jiangxi, and Fujian provinces, culturally, economically, and socially. Albeit a decrease in the tuberculosis epidemic in recent years, Shantou remains one of the cities with the highest incidence of tuberculosis in China.

### Data

The incidence rate of tuberculosis in Shantou City from 2018 to 2021 was provided by the Shantou Tuberculosis Prevention and Control Institute. The Institute provided daily tuberculosis surveillance data, including details such as the daily reported case count, tuberculosis incidence per day, age, gender, occupation, and treatment status, spanning from January 1st, 2018, to December 31st, 2021. The diagnosis of tuberculosis was based on the “Diagnostic criteria for Tuberculosis” released by Ministry of Health in China in 2017. The diagnostic criteria include epidemiological history, clinical findings, chest imaging, laboratory tests, and bronchoscopy.

### Statistical analysis

The tuberculosis incidence rate for each year from 2018 to 2021 was calculated using the recommended method by the Shantou Tuberculosis Prevention and Control Institute. The specific calculation formulas are as follows:

$$IR[n]\; = \;\frac{{Number\;of\;tuberculosis\;cases\;in\;that\;year}}{{Total\;population\;of\;that\;year\;}}$$

$$The\;total\;population[n]\; = \;$$

$$\frac{\begin{array}{l}(Population\;at\;the\;end\;of\;the\;year\; + \\\;Population\;at\;the\;beginning\;of\;the\;year)\end{array}}{2}$$

Where IR represents the tuberculosis incidence rate, and n represents the year (2018, 2019, 2020, 2021). For the number of tuberculosis cases, we include clinically diagnosed cases and confirmed cases. According to the tuberculosis diagnosis guidelines, clinically diagnosed cases refer to those that meet one of the following conditions without microbiological confirmation: (1) Three negative sputum smear examinations, with chest imaging showing lesions consistent with active pulmonary tuberculosis and accompanied by suspected symptoms such as cough, sputum, and hemoptysis; (2) Three negative sputum smear examinations, with chest imaging showing lesions consistent with active pulmonary tuberculosis and a strong positive tuberculin skin test; (3) Three negative sputum smear examinations, with chest imaging showing lesions consistent with active pulmonary tuberculosis and a positive anti-tuberculosis antibody test; (4) Three negative sputum smear examinations, with chest imaging showing lesions consistent with active pulmonary tuberculosis and histopathological examination of extrapulmonary tissues confirming tuberculosis lesions; (5) Suspected pulmonary tuberculosis with three negative sputum smear examinations, which can exclude other lung diseases after diagnostic treatment or follow-up observation. Confirmed cases refer to pulmonary tuberculosis cases diagnosed based on microbiological evidence, including smear-positive pulmonary tuberculosis, culture-positive pulmonary tuberculosis, and cases with pulmonary lesions confirmed by pathological examination.

We divided the data into two periods: prior to the pandemic (January 1st, 2018, to December 31st, 2019) and during the pandemic (January 1st, 2020, to December 31st, 2021). The tuberculosis incidence rate for every two years was represented by the average of the tuberculosis incidence rates for those two years. To better understand the impact of the pandemic on tuberculosis, we also obtained data from 2014 to 2017, with each two-year period calculated separately for 2014–2015, 2016–2017, 2018–2019, and 2020–2021 in Shantou City. The calculation method is the same as above.

Interrupted Time Series (ITS) is a method used to assess whether interventions have an impact on outcome indicators by comparing outcome variables and regression line slopes before and after interventions [17]. In this study, we employed ITS to analyze the incidence of tuberculosis prior to and during the COVID-19 pandemic. The specific model is as follows:

$${Y_t} = {\rm{ }}{\beta _0} + {\rm{ }}{\beta _1}{X_1} + {\rm{ }}{\beta _2}{X_2} + {\rm{ }}{\beta _3}{X_3} + {\rm{ }}\varepsilon t$$

Where Y_t_ represents the monthly incidence of tuberculosis; β_0_ represents the baseline level of tuberculosis incidence prior to the pandemic; β_1_ represents the long-term trend of tuberculosis incidence prior to the pandemic; β_2_ represents the immediate change in tuberculosis incidence after the pandemic; β_3_ estimates the slope change in tuberculosis incidence after the pandemic. ε_t_ represents the random error term. X_1_ is the time elapsed from the beginning to the end of the study, in months (values 0, 1, 2, …, 47, totaling 48 months); X_2_ is the impact of the pandemic (value 0 for unaffected by the pandemic, 1 for affected by the pandemic); X_3_ is the time of the impact of the pandemic (value 0 for unaffected by the pandemic, values 1, 2, … for affected by the pandemic).

All analyses were performed using R (version 4.2.0) packages “stats”, “ggplot2”, and “reshape”.

## Results

We collected a total of 21,503 tuberculosis cases in Shantou, China between 2018 and 2021, with the corresponding incidence rate detailed in Table S1. Among them, 13,414 cases occurred prior to the pandemic (2018–2019), and 8,089 cases occurred during the pandemic (2020–2021). Overall, the average incidence of tuberculosis decreased by 32.45% during the pandemic as compared to that prior to the pandemic. There is a significant difference (*p* < 0.05) in the incidence of tuberculosis between the two periods.

**Fig. 1:**
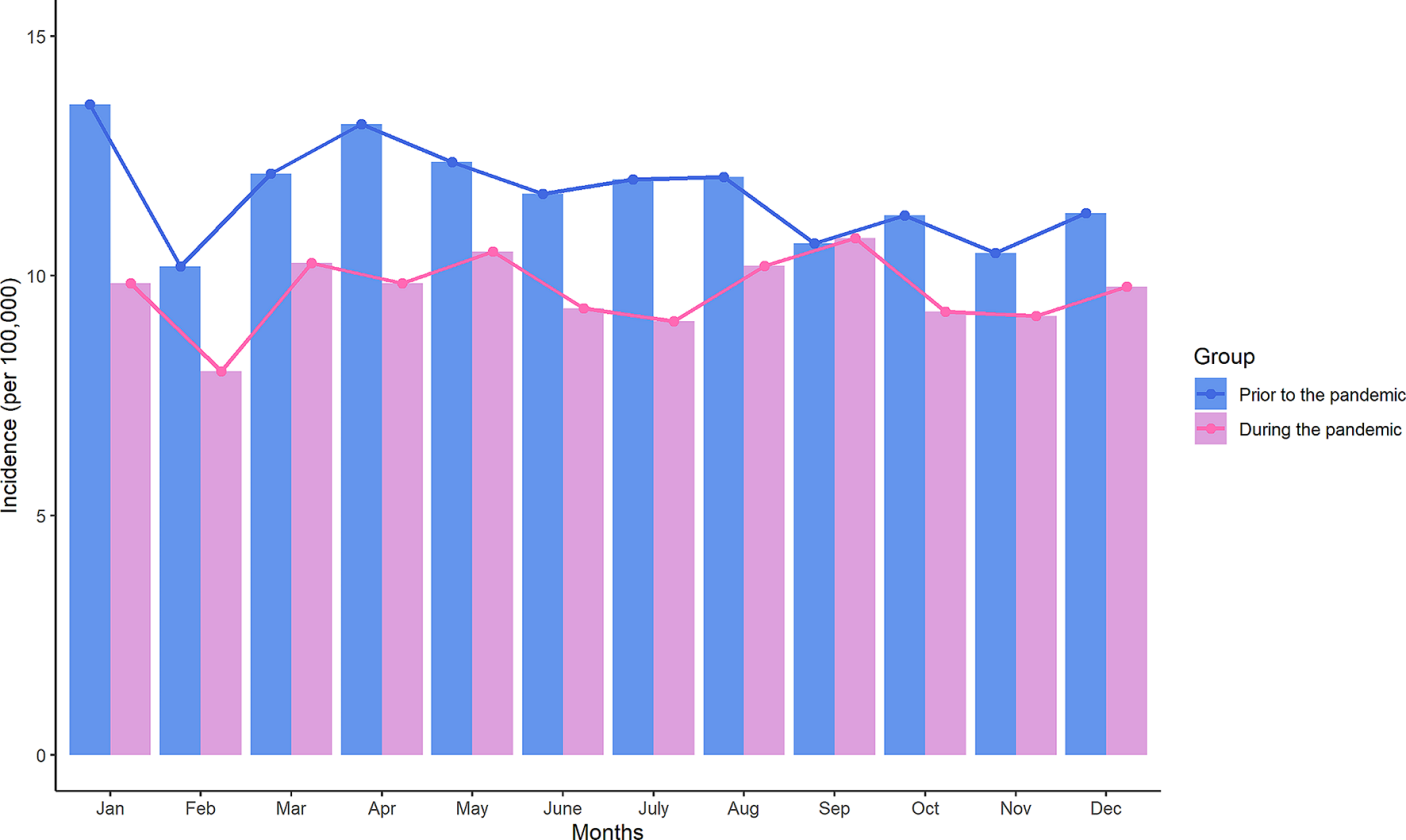
Incidence of tuberculosis cases prior to and during the COVID-19 pandemic in Shantou, China

**Fig. 2:**
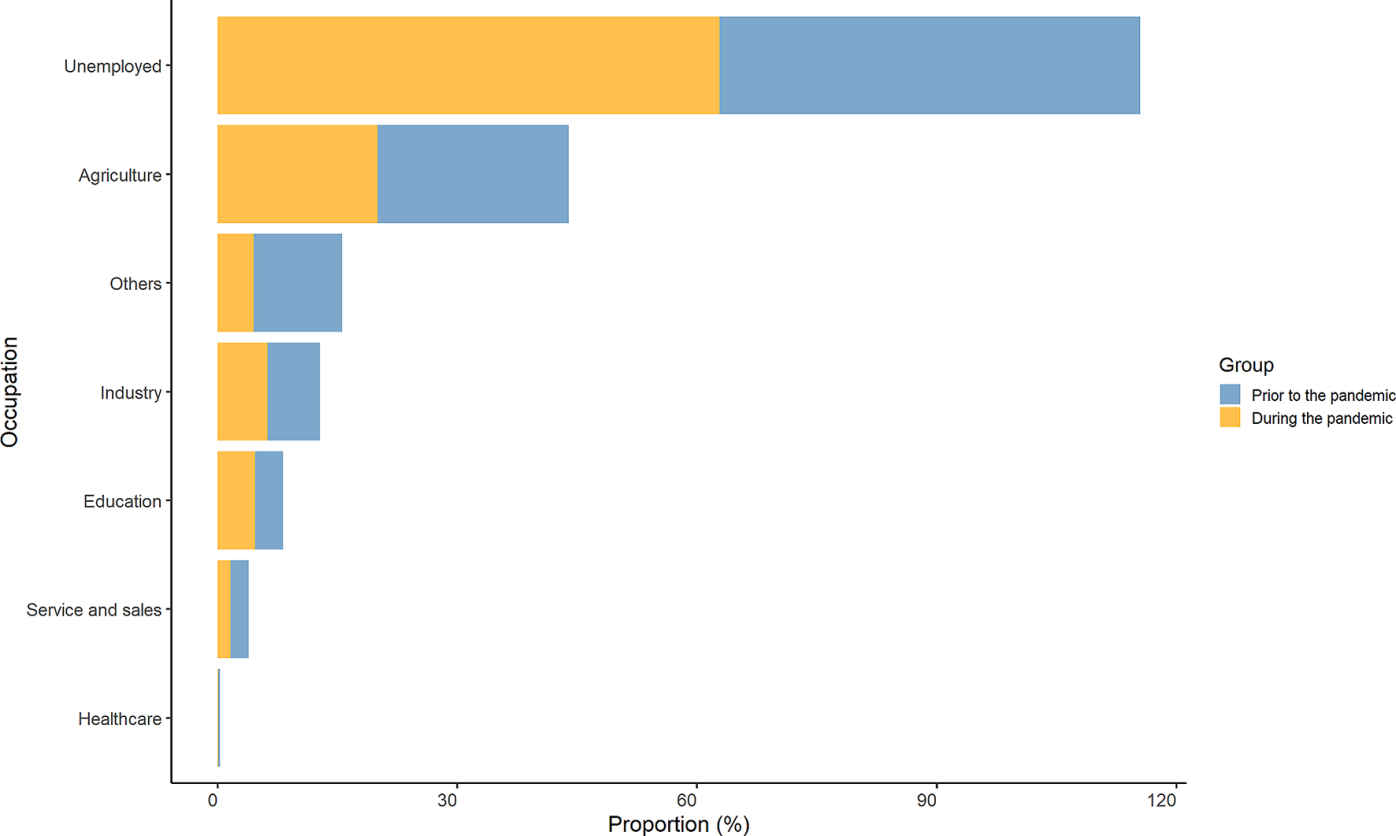
The proportion of tuberculosis incidence by occupation prior to and during the COVID-19 pandemic in Shantou, China

Figure 1 depicts the variation in tuberculosis incidence in different months prior to and during the pandemic. We observed that the incidence of tuberculosis significantly decreased during the pandemic compared to the pre-pandemic period for each month. It is noteworthy that, compared to the pre-pandemic period, the incidence of tuberculosis in all age groups aged 25 and above has significantly decreased during the pandemic (Table S2). The incidence of patients under the age of 25 did not change significantly between the two periods. Figure 2 presents the distribution of tuberculosis incidence in different occupations prior to and during the pandemic in Shantou, China. Each occupation is depicted using two stacked bar charts, with blue representing the pre-pandemic period and orange representing the pandemic period. Our findings indicate a notable reduction in tuberculosis incidence rates among occupations such as agriculture, joblessness (unemployed group), and industrial sectors during the pandemic, with the most substantial decline observed in the unemployed group.

**Fig. 3:**
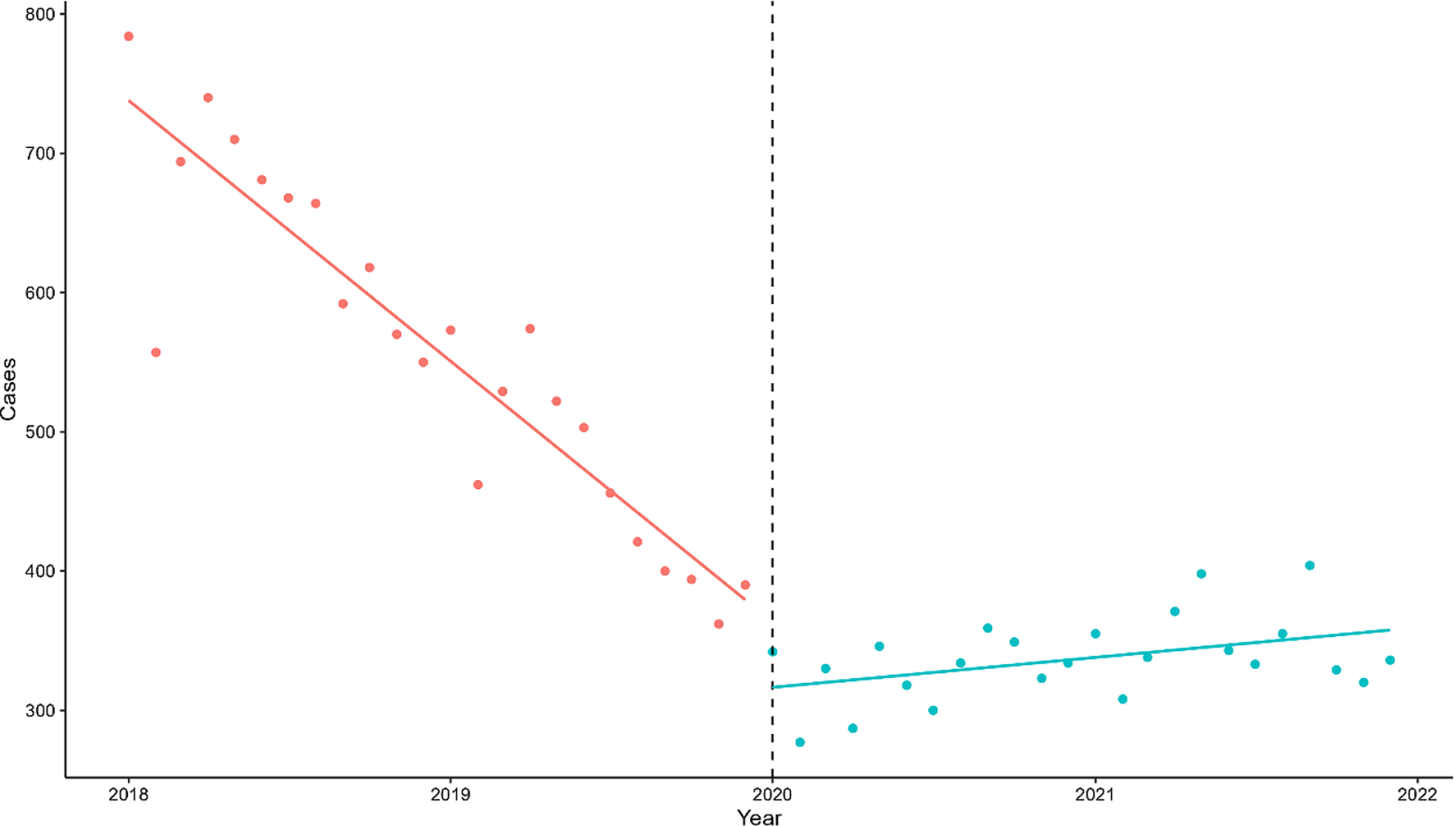
Results of interrupted time series prior to and during the COVID-19 pandemic

ITS indicate a significant decrease in tuberculosis incidence during the outbreak of the COVID-19 pandemic compared to pre-pandemic levels (β_2_=-47.57, *p* < 0.05). During the COVID-19 pandemic, the number of tuberculosis cases exhibited a monthly increasing trend, and the growth rate of cases post-pandemic increased compared to before (β_3_ = 17.38, *p* < 0.05). (Figure. 3)

**Fig. 4:**
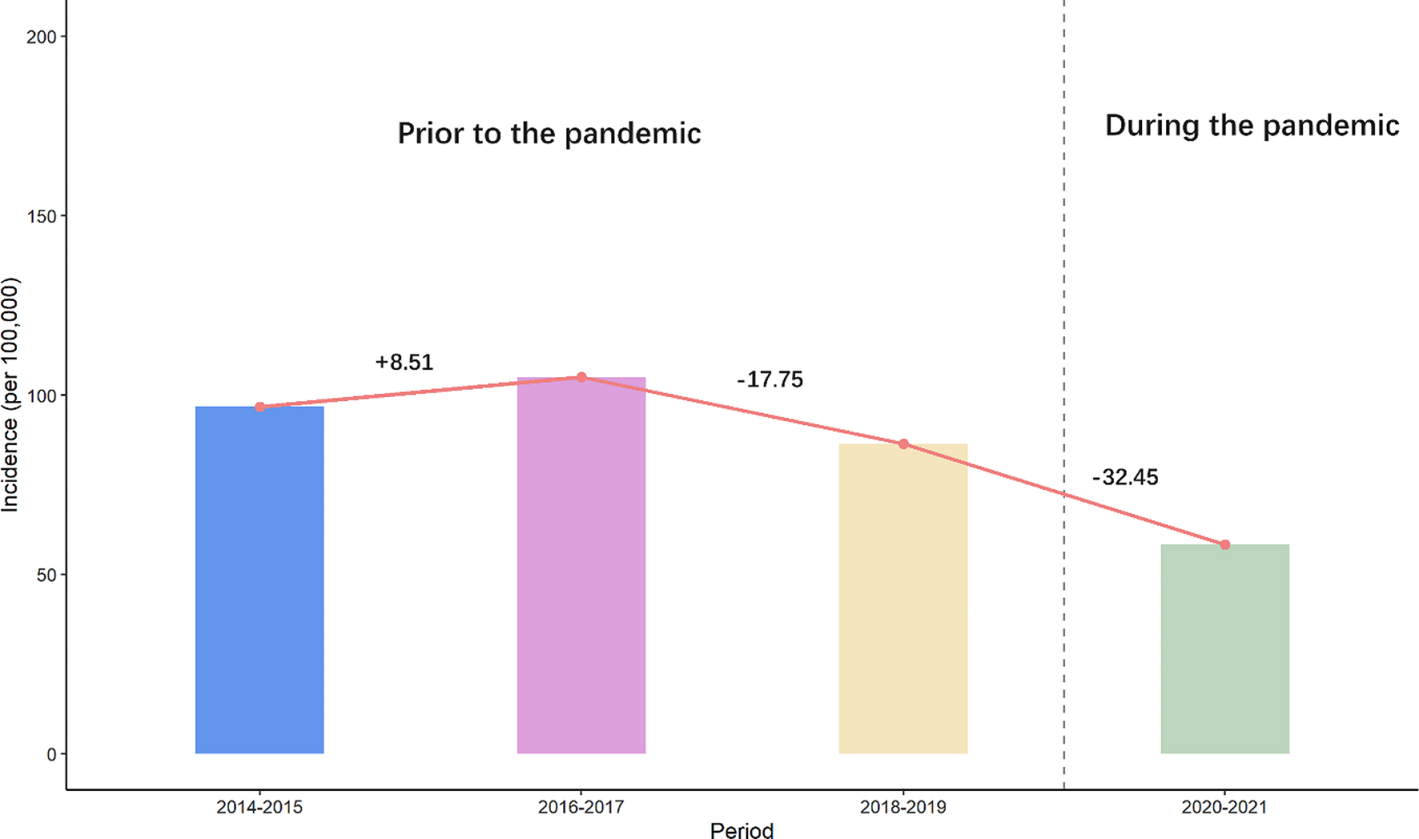
Two-year incidence and trend of tuberculosis in Shantou, China during 2014–2021

To better understand the impact of the pandemic on the incidence of tuberculosis, we included longer pre-pandemic data (extending to 2014) for its comparison with the incidence during the pandemic. Figure 4 shows the biennial incidence of tuberculosis between 2014 and 2017. Overall, the tuberculosis incidence was on the rise (8.51% increase in incidence for the first periods), but during the pandemic the incidence decreased by 32.45%. According to the ITS, the incidence of tuberculosis prior to the pandemic was significantly higher than that during the pandemic (*p* < 0.05). We also aggregated all the pre-pandemic data together to calculate the incidence rate instead of using the two-year incidence rate, and the pre-pandemic incidence was still significantly higher than during the pandemic period (data not shown).

## Discussion

Our study utilized ITS analyses to assess the impact of measures taken to address the COVID-19 pandemic on the incidence of tuberculosis in Shantou, China. Overall, we found a significant decline in the incidence of tuberculosis during the pandemic. The stratified analysis showed that a significant drop in the incidence of tuberculosis among middle-aged and elderly individuals was observed during the pandemic. Due to the middle-aged people are the backbone of social productivity, and the elderly generally have other underlying diseases, they warrant an extra attention. The government may consider monitoring and managing middle-aged and elderly people more closely, like taking measures to restrict them from participating in large-scale gathering activities or wearing masks when going out on a daily basis. In terms of the occupational distribution, the unemployed and agricultural populations experienced the most significant declines in the incidence of tuberculosis during the pandemic. This may be due to the fact that the unemployed and those live in rural areas move relatively less therefore they have less chance of being infected.

Our study was preceded by several articles discussing the impact of the COVID-19 pandemic on tuberculosis, some of which observed trends similar to ours [5, 18]. For instance, the incidence rate of tuberculosis in Serbia declined from 9/100,000 in 2019 to 4.5/100,000 in 2020 [19]. Meanwhile, an American study observed that the reported cases of tuberculosis in 2020 has dropped by 20% compared with those in 2019 [20]. Additionally, a cross-sectional study in Shanghai found that the detection rate of tuberculosis significantly decreased during the COVID-19 pandemic [21]. After analyzing these pieces of literature, we discovered that the incidence of tuberculosis decreased to varying degrees in different countries during the COVID-19 pandemic. We speculate that these variations in the incidence of tuberculosis may be attributed to the differences in the approaches taken by various countries to combat the pandemic. For example, Sweden did not implement comprehensive lockdown measures but adopted a looser prevention and control strategy during the COVID-19 pandemic. The Swedish government emphasizes personal responsibility and voluntary compliance with public health measures, including social distancing, promoting the wearing of masks, and strengthening cleaning and disinfection measures [22]. In contrast, the Italian government implemented stringent lockdown measures, including restricting the movement of people, closing schools and businesses, and prohibiting large-scale activities in response to the pandemic.

At present, there is insufficient evidence to prove a direct causal relationship between the pandemic and the decline in the incidence of tuberculosis. The observed association between the pandemic and the decline may be explained by several speculative reasons. For example, in response to the pandemic, China issued the “Notice on the Prevention and Control of the Novel Coronavirus Pneumonia Pandemic” on January 26th, 2020. This notice emphasized the importance of personal protection and introduced specific measures such as wearing masks and practicing frequent handwashing. These measures aimed at minimizing the spread of the coronavirus may also reduce the spread of MTB and thus reduce the incidence of tuberculosis. More specifically, as the modes of transmission for COVID-19 and tuberculosis are similar, the preventive measures implemented to contain the spread of COVID-19 can also inadvertently contribute to preventing the transmission of tuberculosis. Other measures, such as non-contact services in public and commercial places, bans on public transport services, and restrictions on population movement have played a similar role.

While enforced measures to restrict population movement have proven effective in slowing the propagation of COVID-19, they may inadvertently lead to undesirable outcomes such as impeding timely medical care for those suffering from tuberculosis. This presents onerous challenges in the identification of tuberculosis cases due to limited transportation means and a decrease in medical visits. During the COVID-19 pandemic, the usage of public transportation in some cities has decreased. For instance, in cities like Italy and Spain, the usage of public transportation has decreased by 95% compared to the early stages of the pandemic [23]. According to a study conducted in Poland, global road transportation volume decreased by over 50% by the end of March 2020 compared to the same period in 2019. Similarly, compared to January 2020, public transport passenger volume decreased by 77% [24]. Another study in the same region also indicated a 66% reduction in individual travel time across all age groups during the COVID-19 period [23]. Besides, a study in Uganda found that through interviews with tuberculosis patients, 79.9% of them believed that the COVID-19 pandemic led to transportation restrictions, such as a lack of access to transportation to medical facilities, insufficient funds to cover transportation costs, and long distances to medical facilities, severely limiting their opportunities to access tuberculosis care services at treatment units [25]. On another note, the reassignment of healthcare resources and services and the prioritization of disease surveillance and reporting systems might have hindered the reporting of new cases. For instance, a retrospective study in South Africa found that during the COVID-19 pandemic, the utilization rate of primary healthcare services dropped significantly to 10.53% compared to previous periods [26]. A study in Pakistan similarly reached a similar conclusion [27]. Throughout the pandemic, the government focused predominantly on COVID-19 prevention and control, mandating all localities to enhance the storage and deployment of medical supplies like masks, goggles, and protective clothing to match the requirements for epidemic management. These measures could potentially adversely impact the detection and treatment of tuberculosis, as resource allocation has shifted towards addressing COVID-19, affecting the availability and accessibility of resources and services related to controlling tuberculosis.

Moreover, the relocation of healthcare personnel considerably reduced the workforce available for tuberculosis control, potentially leading to service interruptions like tuberculosis testing and rapid diagnosis, culminating in situations where tuberculosis patients cannot be diagnosed or treated promptly after arriving at the hospital. For example, a study in Turkey found that during the pandemic, there was a significant decrease in the incidence of tuberculosis, the number of screenings, and contact tracing efforts. It was noted that this trend might be attributed to the redirection of resources towards controlling COVID-19, which impacted the available workforce for tuberculosis control during the COVID-19 period. Hence, the decline witnessed in tuberculosis incidence amid the pandemic may not be legitimate but rather a consequence of inadequate reporting. To substantiate this hypothesis, a surge in tuberculosis visits after the pandemic would serve as confirmation, and it is imperative to conduct further investigations for validation.

Our study comes with a few limitations. Firstly, the quantity of tuberculosis cases gathered was dependent on a passive monitoring system, which could give rise to under-reporting and an undervaluation of actual tuberculosis patients. Secondly, despite the COVID-19 pandemic persisting, we designated the period of the pandemic as 2020–2021, disregarding the impact of its later stages. Thirdly, intervention measures and epidemics could differ across regions, making it challenging to directly extrapolate research outcomes from one area to another. Lastly, responses to the pandemic were diverse and complex, involving multiple levels and numerous measures, such as quarantines, medical resource allocation, and personnel movement restrictions. These policies may interconnect and mutually influence each other, making it challenging to attribute their effects solely to changes in the incidence of tuberculosis cases.

## Conclusion

To sum up, we have observed a significant drop in the incidence of tuberculosis in Shantou, China during the pandemic compared to the pre-pandemic period. This decline can be attributed to population-level interventions such as community lockdowns, personal quarantines, and disease prevention and control campaigns. These measures are objectively effective in lowering the incidence of tuberculosis. However, it should be noted that this decrease may not be an actual reduction in incidence but rather under-reporting. Conversely, treatment delay caused by limited transportation options and medical resource diversion during the pandemic may increase the tuberculosis burden instead. Furthermore, it is important to note that the findings of this study are confined to Shantou, China. Therefore, caution should be exercised when extrapolating the research results to a national scale, as variables in other regions may differ from those in Shantou. Consequently, explanations and inferences regarding the decline in tuberculosis incidence in Shantou require further validation and comparison through nationwide studies.

### Electronic supplementary material

Below is the link to the electronic supplementary material.

Supplementary Material 1

## Data Availability

The datasets used during the current study are available from the corresponding author upon reasonable request.

## References

[1] Ortiz WJ, McKowen RL, Cervantes M (2023). Pulmonary mycetoma with a concomitant reactivation of pulmonary tuberculosis infection: a Case Report and Clinical Pathological Review. Cureus.

[2] Light RW (2010). Update on tuberculous pleural effusion. Respirology.

[3] Turner RD, Bothamley GH (2015). Cough and the transmission of tuberculosis. J Infect Dis.

[4] Lee M-Y, Lin K-D, Hsu W-H, Chang H-L, Yang Y-H, Hsiao P-J (2015). Statin, Calcium Channel Blocker and Beta Blocker Therapy May decrease the incidence of tuberculosis infection in Elderly Taiwanese patients with type 2 diabetes. IJMS.

[5] Sanyaolu A, Okorie C, Hosein Z, Patidar R, Desai P, Prakash S (2021). Global pandemicity of COVID-19: Situation Report as of June 9, 2020. Infect Dis (Auckl).

[6] Arsenault C. COVID-19 and resilience of healthcare systems in ten countries. Nat Med. 2022;28. 10.1038/s41591-022-01750-1 PMC9205770 35288697

[7] Gianella C, Gideon J, Romero MJ (2021). What does COVID-19 tell us about the Peruvian health system?. Can J Dev Stud / Revue Canadienne d’études du développement.

[8] Huang H, Lin C, Liu X, Zhu L, Avellán-Llaguno RD, Lazo MML (2022). The impact of air pollution on COVID-19 pandemic varied within different cities in South America using different models. Environ Sci Pollut Res.

[9] Kaye AD, Okeagu CN, Pham AD, Silva RA, Hurley JJ, Arron BL (2021). Economic impact of COVID-19 pandemic on healthcare facilities and systems: international perspectives. Best Pract Res Clin Anaesthesiol.

[10] Duarte R, Aguiar A, Pinto M, Furtado I, Tiberi S, Lönnroth K (2021). Different disease, same challenges: social determinants of tuberculosis and COVID-19. Pulmonology.

[11] Yang C, An S, Qiao B, Guan P, Huang D, Wu W (2022). Exploring the influence of COVID-19 on the spread of hand, foot, and mouth disease with an automatic machine learning prediction model. Environ Sci Pollut Res.

[12] Li X, Qiao Y, Zhu J, Shi L, Wang Y (2017). The APEC blue endeavor: causal effects of air pollution regulation on air quality in China. J Clean Prod.

[13] Geng M-J, Zhang H-Y, Yu L-J, Lv C-L, Wang T, Che T-L (2021). Changes in notifiable infectious disease incidence in China during the COVID-19 pandemic. Nat Commun.

[14] Soo RJJ, Chiew CJ, Ma S, Pung R, Lee V (2020). Decreased influenza incidence under COVID-19 Control measures, Singapore. Emerg Infect Dis.

[15] Katsumata N, Harama D, Toda T, Sunaga Y, Yoshizawa M, Kono Y (2021). Prevention measures for COVID-19 and changes in Kawasaki Disease incidence. J Epidemiol.

[16] McQuaid CF, Vassall A, Cohen T, Fiekert K, White RG, Covid/Tb Modelling Working Group * (2021). The impact of COVID-19 on TB: a review of the data. int j Tuberc lung dis.

[17] Hategeka C, Ruton H, Karamouzian M, Lynd LD, Law MR (2020). Use of interrupted time series methods in the evaluation of health system quality improvement interventions: a methodological systematic review. BMJ Glob Health.

[18] Daneshvar P, Hajikhani B, Sameni F, Noorisepehr N, Zare F, Bostanshirin N (2023). COVID-19 and Tuberculosis coinfection: an overview of case reports/case series and meta-analysis of prevalence studies. Heliyon.

[19] Pavlovic JM, Pesut DP, Stosic MB (2021). Influence of the COVID-19 pandemic on the incidence of tuberculosis and influenza. Rev Inst Med trop S Paulo.

[20] Deutsch-Feldman M, Pratt RH, Price SF, Tsang CA, Self JL. Tuberculosis — United States, 2020. 2021;70. 10.15585/mmwr.mm7012a1 PMC7993554 33764959

[21] Wu Z, Chen J, Xia Z, Pan Q, Yuan Z, Zhang W (2020). Impact of the COVID-19 pandemic on the detection of TB in Shanghai, China. int j Tuberc lung dis.

[22] Yan B, Zhang X, Wu L, Zhu H, Chen B. Why do countries respond differently to COVID-19? A comparative study of Sweden, China, France, and Japan. American Review of Public Administration.

[23] Lockdowned (2021). Everyday mobility changes in response to COVID-19. J Transp Geogr.

[24] Wielechowski M, Czech K, Grzęda Ł (2020). Decline in mobility: Public Transport in Poland in the time of the COVID-19 pandemic. Economies.

[25] Bbuye M, Muyanja SZ, Sekitoleko I, Padalkar R, Robertson N, Helwig M (2024). Patient level barriers to accessing TB care services during the COVID-19 pandemic in Uganda, a mixed methods study. BMC Health Serv Res.

[26] Heunis C, Chikobvu P, Muteba M, Kigozi-Male G, Engelbrecht M, Mushori P (2023). Impact of COVID-19 on selected essential public health services – lessons learned from a retrospective record review in the Free State, South Africa. BMC Health Serv Res.

[27] Baloch AA, Baig N, Baloch F, Suhag Z (2021). Impact on the utilization of Reproductive, maternal, Newborn and Child Health Care Services at Primary Health Care Level during First Wave of COVID-19 outbreak in Pakistan. Cureus.

