# Supplementary material for: Impact of COVID-19 pandemic responses on tuberculosis incidence: insights from Shantou, China
Source: BMC Public Health. 2024 May 30;24:1454. doi: 10.1186/s12889-024-18956-2 (PMC11140913; doi:10.1186/s12889-024-18956-2)
Supplement: Supplementary file 1 — Supplementary Material 1 [file 12889_2024_18956_MOESM1_ESM.docx]

Table S1

Incidence of tuberculosis in Shantou, China, during 2018-2019 and 2020-2021.

| Period | Incidence  (per 100,000) | Number of Cases | Mean | SD^*^ | Min^*^ |  | Percentile | | | Max^*^ | p-value |
| --- | --- | --- | --- | --- | --- | --- | --- | --- | --- | --- | --- |
|  |  |  |  |  |  | 25^th^ | | 50^th^ | 75^th^ |  |  |
| 2018-2019 | 86.36 | 13,414 | 19.18 | 9.79 | 1.5 | 17.5 | | 18 | 13 | 71 | <0.05 |
| 2020-2021 | 58.36 | 8,089 | 16.09 | 7.9 | 2 | 15 | | 15 | 11 | 64 |  |

^*^SD=Standard deviation; Min=Minimum; Max=Maximum


Table S2

Number of tuberculosis cases stratified by sex, age and occupation in Shantou, China, during 2018-2019 and 2020-2021.

| Characteristics | Periods | |
| --- | --- | --- |
|  | 2018-2019 | 2020-2021 |
| Gender |  |  |
| Male | 10,034 | 5,933 |
| Female | 3,380 | 2,167 |
| Total | 1,3414 | 8,089 |
| Age group |  |  |
| 0-4 | 16 | 2 |
| 5-14 | 52 | 44 |
| 15-24 | 1,436 | 859 |
| 25-44 | 2,628 | 1,685 |
| 45-64  65+ | 5,725  3,557 | 3,329  2,170 |
| Total | 13,414 | 8,089 |
| Months |  |  |
| January | 1,357 | 697 |
| February | 1,019 | 585 |
| March | 1,223 | 668 |
| April | 1,314 | 658 |
| May | 1,232 | 744 |
| June | 1,184 | 661 |
| July | 1,124 | 633 |
| August  September | 1,085  992 | 689  763 |
| October | 1,012 | 678 |
| November | 932 | 643 |
| December | 940 | 670 |
| Total | 13,414 | 8,089 |
| Occupation |  |  |
| Agriculture | 3,208 | 1,567 |
| Service and sales | 304 | 126 |
| Unemployed | 7,053 | 4,917 |
| Industry | 876 | 490 |
| Healthcare | 28 | 5 |
| Education  Others | 468  1,477 | 367  354 |
| Total | 13,414 | 8,089 |
